# Supplementary material for: Bond-Orbital-Resolved Piezoelectricity in Sp2-Hybridized Monolayer Semiconductors
Source: Materials (Basel). 2022 Nov 4;15(21):7788. doi: 10.3390/ma15217788 (PMC9653688; doi:10.3390/ma15217788)
Supplement: Supplementary file 1 [file materials-15-07788-s001.zip › materials-1977849-supplementary.pdf]

Supplementary Materials for "Bond-Orbital-Resolved Piezoelectricity in Sp^2-Hybridized Monolayer Semiconductors"

Zongtan Wang [1], Yulan Liu [1,*] and Biao Wang [2,*]

[1]School of Aeronautics and Astronautics, Sun Yat-sen University, Shenzhen 518000, China

[2]Sino-Franch Institute of Nuclear Engineering and Technology, Sun Yat-sen University, Zhuhai 519082, China

* Correspondence: stslyl@mail.sysu.edu.cn (Y.L.); wangbiao@mail.sysu.edu.cn (B.W.)

S1. The PBC of the π-valence band in the TB model

In this section, we present the detailed steps needed to obtain the expression of $\Omega^{\pi}_{2,22}(\Delta,\boldsymbol{k})$ in Equation (9) from Equation (8); i.e.:

$$\Omega^{\pi}_{i,jk}(\boldsymbol{k}) = i\frac{\langle u_7^0 | v_i^{\pi} | u_8^0\rangle\langle u_8^0 | \tilde{v}_{jk}^{\pi} | u_7^0\rangle - c.c.}{(E_7^0 - E_8^0)^2} \tag{S1}$$

We start from the π-band TB Hamiltonian in Equation (4) in the main text. By setting $\epsilon_p^A = \pm\Delta, (\epsilon_p = 0)$, it can be rewritten as:

$$H^{\pi}(\boldsymbol{k},\boldsymbol{\varepsilon}) = \begin{bmatrix} \Delta/2 & f(\boldsymbol{k},\boldsymbol{\varepsilon}) \\ f^*(\boldsymbol{k},\boldsymbol{\varepsilon}) & -\Delta/2 \end{bmatrix}. \tag{S2}$$

When $\boldsymbol{\varepsilon} = \boldsymbol{0}$, we have $H^{\pi}(\boldsymbol{k}) = H^{\pi}(\boldsymbol{k},\boldsymbol{0})$, and its eigenvalue and eigenvector for the valence ($n = 7$) and conduction ($m = 8$) band are given as:

$$\begin{aligned} E^0_{7(8)} &= \pm\sqrt{|f(\boldsymbol{k})|^2 + \Delta^2/4}, \\ |u^0_{7(8)}\rangle &= \frac{1}{\sqrt{2E_8^0}}\begin{bmatrix} \dfrac{f(\boldsymbol{k})}{\sqrt{E_8^0 \mp \Delta/2}} \\ \pm\sqrt{E_8^0 \mp \Delta/2} \end{bmatrix}. \end{aligned} \tag{S3}$$

The remaining two terms in Equation (S1) can be calculated as:

$$v_i^{\pi} = \frac{\partial H^{\pi}(\boldsymbol{k})}{\partial k_i} = at\begin{bmatrix} 0 & g_i \\ g_i^* & 0 \end{bmatrix}, \tag{S4}$$

with

$$\begin{aligned} g_1 &= -i\sqrt{3}(e^{-i\boldsymbol{k}\cdot\boldsymbol{\delta}_2} - e^{-i\boldsymbol{k}\cdot\boldsymbol{\delta}_3})/2, \\ g_2 &= i(2e^{-i\boldsymbol{k}\cdot\boldsymbol{\delta}_1} - e^{-i\boldsymbol{k}\cdot\boldsymbol{\delta}_2} - e^{-i\boldsymbol{k}\cdot\boldsymbol{\delta}_3})/2, \end{aligned} \tag{S5}$$

and

$$\tilde{v}_{jk}^{\pi} = \frac{\partial H^{\pi}(\boldsymbol{k},\boldsymbol{\varepsilon})}{\partial \varepsilon_{jk}} = \beta_{pp\pi} t \begin{bmatrix} 0 & h_{jk} \\ h_{jk}^{*} & 0 \end{bmatrix}, \tag{S6}$$

with

$$\begin{aligned} h_{11} &= -3(e^{-i\boldsymbol{k}\cdot\boldsymbol{\delta}_2} + e^{-i\boldsymbol{k}\cdot\boldsymbol{\delta}_3})/4, \\ h_{22} &= -(4e^{-i\boldsymbol{k}\cdot\boldsymbol{\delta}_1} + e^{-i\boldsymbol{k}\cdot\boldsymbol{\delta}_2} + e^{-i\boldsymbol{k}\cdot\boldsymbol{\delta}_3})/4, \\ h_{12} &= -\sqrt{3}(e^{-i\boldsymbol{k}\cdot\boldsymbol{\delta}_2} - e^{-i\boldsymbol{k}\cdot\boldsymbol{\delta}_3})/2. \end{aligned} \tag{S7}$$

Substituting Equation (S3-S7) into Equation (S1), the PBC for the π-valence band can be obtained as:

$$\Omega_{i,jk}^{\pi} = i\frac{\beta_{pp\pi} a t^2 \Delta (g_i^* h_{jk} - g_i h_{jk}^*)}{8(E_8^0)^3}. \tag{S8}$$

For $i = j = k = 2$, using the Euler's formula $e^{-i\boldsymbol{k}\cdot\boldsymbol{\delta}_I} = \cos(\boldsymbol{k}\cdot\boldsymbol{\delta}_I) - i\sin(\boldsymbol{k}\cdot\boldsymbol{\delta}_I)$, we can express $\Omega_{2,22}^{\pi}$ as the explicit function of Δ and $\boldsymbol{k}$ as the following:

$$\Omega_{2,22}^{\pi}(\Delta,\boldsymbol{k}) = \frac{\beta_{pp\pi} a t^2 \Delta}{4(E_8^0)^3}\left[\cos\left(\boldsymbol{k}\cdot\frac{\boldsymbol{\delta}_2-\boldsymbol{\delta}_3}{2}\right)\cos\left(\boldsymbol{k}\cdot\frac{\boldsymbol{\delta}_3-\boldsymbol{\delta}_1}{2}\right)\cos\left(\boldsymbol{k}\cdot\frac{\boldsymbol{\delta}_1-\boldsymbol{\delta}_2}{2}\right)-1\right] \tag{S9}$$

Note that $\Delta = \mathrm{sgn}(\Delta)|\Delta| = C_v|\Delta|$; this is just Equation (9) in the main text.

S2. The relation between the PBC and MBC of the π-valence band in the valley model

To prove the relation given in Equation (13) for the valley model, we then repeat the above derivation of PBC from the massive Dirac Hamiltonian $H^{\tau}(\boldsymbol{q},\boldsymbol{\varepsilon})$ in Equation (12) in the main text, the eigenvalue and eigenvector of which, in the absence of strain, are given as:

$$\begin{aligned} E_{7(8)}^{\tau} &= \pm\sqrt{(\hbar v_F|\boldsymbol{q}|)^2 + \Delta^2/4}, \\ |u_{7(8)}^{\tau}\rangle &= \frac{1}{\sqrt{2E_8^{\tau}}}\begin{bmatrix} \dfrac{\hbar v_F(\tau q_1 - i q_2)}{\sqrt{E_8^{\tau} \mp \Delta/2}} \\ \pm\sqrt{E_8^{\tau} \mp \Delta/2} \end{bmatrix}. \end{aligned} \tag{S10}$$

Since $H^{\tau}(\boldsymbol{q},\boldsymbol{\varepsilon})$ is linear in both q_i and ε_{jk} (or equally, $\tilde{A}_l = \beta_{pp\pi}/2a\sum_{jk}\gamma_{ljk}\varepsilon_{jk}$), when taking its partial derivative with respect to q_i or ε_{jk}, we are facilitated by a linear mapping relationship $\partial_{\varepsilon_{jk}} \to \partial_{\tilde{A}_l} \to \partial_{q_l}$ to relate $\tilde{v}_{jk}^{\tau} = \partial_{\varepsilon_{jk}} H^{\tau}(\boldsymbol{q},\boldsymbol{\varepsilon})|_{\boldsymbol{\varepsilon}\to\boldsymbol{0}}$ with the

crystal velocity $v_i^\tau = \partial_{q_i} H^\tau(\boldsymbol{q},\boldsymbol{0}) = \hbar v_F \sigma_i^\tau$ (here, $\sigma_i^\tau = \tau^i \sigma_i$). To be specific, using the chain rule of differentiation, we have:

$$\frac{\partial H^\tau(\boldsymbol{q},\boldsymbol{\varepsilon})}{\partial \varepsilon_{jk}} = \sum_l \frac{\partial H^\tau(\boldsymbol{q},\boldsymbol{\varepsilon})}{\partial \tilde{A}_l} \frac{\partial \tilde{A}_l}{\partial \varepsilon_{jk}} = \sum_l \frac{\beta_{pp\pi}\gamma_{ljk}}{2a} \frac{\partial H^\tau(\boldsymbol{q},\boldsymbol{\varepsilon})}{\partial \tilde{A}_l} \tag{S11}$$

and

$$\tau \frac{\partial H^\tau(\boldsymbol{q},\boldsymbol{\varepsilon})}{\partial \tilde{A}_l}\bigg|_{\boldsymbol{\varepsilon}\to\boldsymbol{0}} = -\frac{\partial H^\tau(\boldsymbol{q},\boldsymbol{0})}{\partial q_l}. \tag{S12}$$

As a result:

$$\tilde{v}_{jk}^\tau = -\sum_l \tau \frac{\beta_{pp\pi}\gamma_{ljk}}{2a} v_l^\tau. \tag{S13}$$

This further enables us to reformulate the PBC of the τ -valley as:

$$\begin{aligned}
\Omega_{i,jk}^\tau(\boldsymbol{q}) &= i\frac{\langle u_7^\tau | v_i^\tau | u_8^\tau\rangle\langle u_8^\tau | \tilde{v}_{jk}^\tau | u_7^\tau\rangle - c.c.}{(E_7^\tau - E_8^\tau)^2} \\
&= -\sum_l \tau \frac{\gamma_{ljk}\beta_{pp\pi}}{2a}\left[i\frac{\langle u_7^\tau | v_i^\tau | u_8^\tau\rangle\langle u_8^\tau | \tilde{v}_{jk}^\tau | u_7^\tau\rangle - c.c.}{(E_7^\tau - E_8^\tau)^2}\right] \\
&= -\sum_l \tau \frac{\gamma_{ljk}\beta_{pp\pi}}{2a}\Omega_{il}^\tau(\boldsymbol{q}) \\
&= -\sum_l \tau \frac{\epsilon_{il}\gamma_{ljk}\beta_{pp\pi}}{2a}\Omega_\tau(\boldsymbol{q}),
\end{aligned} \tag{S14}$$

During the last step of this, the rank-2 Levi–Civita tensor ϵ_{il} is introduced to specify the antisymmetric property $\Omega_{il}^\tau(\boldsymbol{q}) = -\Omega_{li}^\tau(\boldsymbol{q})$; $\Omega_\tau(\boldsymbol{q}) = \Omega_{12}^\tau(\boldsymbol{q})$ is just the MBC of the τ -valley, which, by combining $\hbar v_F = 3at/2$, $v_i^\tau = \hbar v_F \tau^i \sigma_i$, and Equation (S10), can be straightforwardly calculated as:

$$\begin{aligned}
\Omega_\tau(\boldsymbol{q}) &= i\tau(\hbar v_F)^2 \frac{\langle u_7^\tau | \sigma_1 | u_8^\tau\rangle\langle u_8^\tau | \sigma_2 | u_7^\tau\rangle - c.c.}{(E_7^\tau - E_8^\tau)^2} \\
&= \tau \frac{9a^2t^2\Delta}{2[(3atq)^2 + \Delta^2]^{3/2}},
\end{aligned} \tag{S15}$$

with $q = |\boldsymbol{q}| = \sqrt{q_1^2 + q_2^2}$. We thus finally obtain Equation (13) in the main text.

S3. The piezoelectric current contributed by the τ -valley of the π-valence band

Here, we give the derivation of the piezoelectric current $\tilde{J}^{\tau}$ in Equation (17) in the main text. For the τ -valley of the π-valence band, the general expression of the piezoelectric current component in Equation (5) is reduced to:

$$\tilde{J}_i^{\tau} = 2e\sum_{jk}\int_{\Lambda} d\boldsymbol{q}\Omega_{i,jk}^{\tau}(\boldsymbol{q})\dot{\varepsilon}_{jk} / (2\pi)^2. \tag{S16}$$

Denoting the in-plane unit direction vectors by $\hat{\boldsymbol{n}}_{i=1,2} = \hat{\boldsymbol{x}}, \hat{\boldsymbol{y}}$ and using the relationship in Equation (S14), we can reformulate the valley-dependent piezoelectric current induced by the in-plane adiabatic strain as:

$$\begin{aligned}\tilde{\boldsymbol{J}}^{\tau} &= \sum_i \tilde{J}_i^{\tau}\hat{\boldsymbol{n}}_i = 2e\sum_{i,jk}\int_{\Lambda}\frac{d\boldsymbol{q}}{(2\pi)^2}\Omega_{i,jk}^{\tau}(\boldsymbol{q})\dot{\varepsilon}_{jk}\hat{\boldsymbol{n}}_i \\ &= -2e\int_{\Lambda}\frac{d\boldsymbol{q}}{(2\pi)^2}\Omega_{\tau}(\boldsymbol{q})\sum_{il,jk}\epsilon_{il}\tau\frac{\beta_{pp\pi}\gamma_{ljk}\dot{\varepsilon}_{jk}}{2a}\hat{\boldsymbol{n}}_i \\ &= (2\hbar/e)\sigma_{\mathrm{H}}^{\tau}\sum_{il}\epsilon_{il}(-\tau\dot{\tilde{A}}_l)\hat{\boldsymbol{n}}_i \\ &= (2\hbar/e)\hat{\boldsymbol{\sigma}}_{\mathrm{H}}^{\tau}\times\tilde{\mathbf{E}}^{\tau}.\end{aligned} \tag{S17}$$

This is Equation (17) in the main text.

S4. The fitting of the TB parameters for the π-bands of unstrained sp^2 crystals

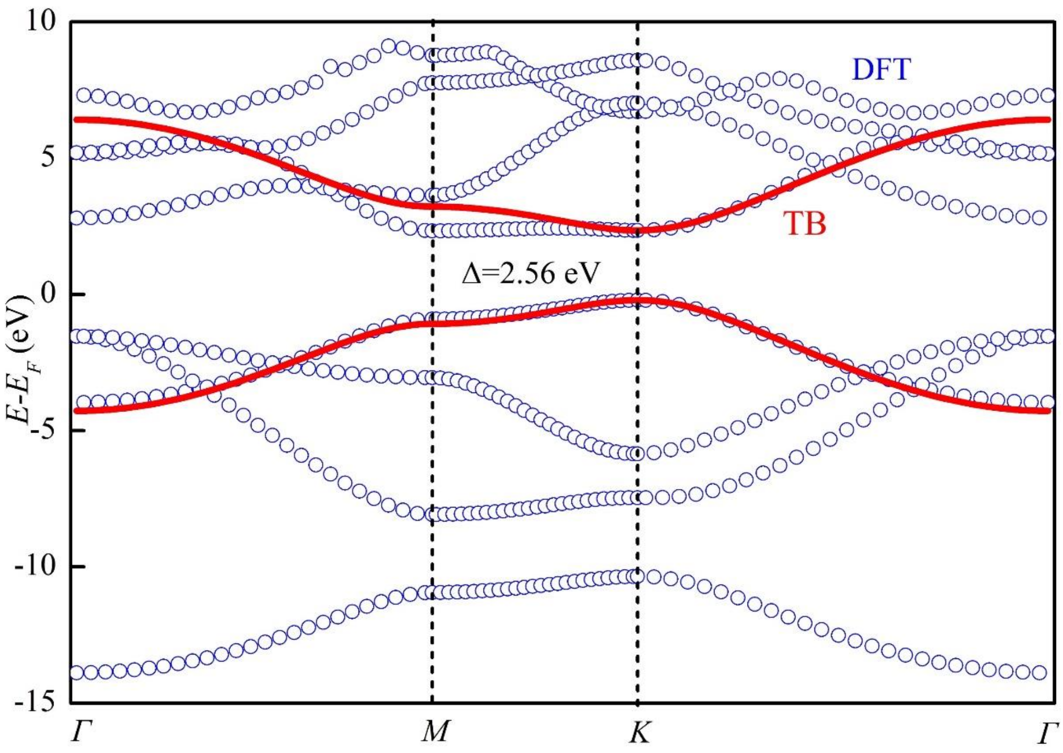

Figure S1. Comparison between the TB band structure (red lines) and the DFT band structure (blue circles) for the h-SiC monolayer. Here, the Fermi energy was set at $E_F = 0$.

In this section, we take h-SiC as an example to illustrate the procedure for fitting the TB parameters Δ and t for the π-bands of the unstrained sp^2 crystals considered in Table 2 in the main text. We first calculated the DFT band structure of h-SiC with the VASP package to produce the target data $E_{7(8)}^{\mathrm{DFT}}(\boldsymbol{k})$ for its π-bands. Then, by taking the DFT calculated bond length $a = 1.78\,\text{Å}$ as an input, we fit the TB π-band energy $E_{7(8)}^{0}(\boldsymbol{k})$ in Equation (S3) to the corresponding DFT data $E_{7(8)}^{\mathrm{DFT}}(\boldsymbol{k})$ using the least-square method. The fitted TB parameters for the π-bands of h-SiC were $\Delta = 2.56\,\mathrm{eV}$ and $t = -1.74\,\mathrm{eV}$. As shown in Figure S1, the TB band structures (red lines) based on these fitted parameters were very consistent with the DFT results (blue circles). The TB parameters for other sp^2 crystals such as h-BAs and BSb given in Table 2 were also fitted in the same way.
